# OP16 “Progress Is Impossible Without Change”: A Case Study To Feasibly Incorporate Environmental Sustainability In Health Technology Assessment

**DOI:** 10.1017/S0266462324000795

**Published:** 2025-01-07

**Authors:** Robert Malcolm, Rebecca Naylor, Melissa Pegg

## Abstract

**Introduction:**

Environmental sustainability and its incorporation in health technology assessment (HTA) is becoming increasingly researched globally. However, this has yet to lead to a significant impact on HTA processes. This research presents a novel case study, demonstrating how available methods can deepen the understanding of evidence-based approaches to healthcare decision-making and support HTA sustainability policy development.

**Methods:**

A decision-analytic model was developed for a digital health technology used to support primary care in the diagnosis and triage of musculoskeletal conditions. The model mapped the potential impact on the care pathway, capturing differences in resource use, including appointments, medications, diagnostic tests, surgical procedures, and other nonpharmacological treatments. The model was populated from a UK perspective and captured both health economic impact and carbon dioxide equivalents (CO_2_e) impact. Additional potential environmental impacts were then considered qualitatively as part of the evaluation.

**Results:**

The health economic modeling approach captures all stages of the patient care pathway and resource use demonstrating its practicality for simultaneously mapping out the carbon impacts. This methodological approach is reproducible, transparent, and provides a standardized tool for use in future carbon-cost-comparison modeling. This would present decision-makers with more complete information. There are some limitations to this approach, such as ambiguity regarding some carbon data estimates used, but it still provides a more useful summary than no estimated quantification.

**Conclusions:**

Adapting HTA will support wider efforts in health systems to reduce environmental impacts. This model can be practically applied to account for both cost and carbon data, facilitating a holistic and environmentally sustainable approach to decision-making. As part of encouraging additional research into the environment, HTA agencies will need to provide “incentives” for companies to undertake this additional research.

